# TaKeTiNa Music Therapy for Outpatient Treatment of Depression: Study Protocol for a Randomized Clinical Trial

**DOI:** 10.3390/jcm13092494

**Published:** 2024-04-24

**Authors:** Ali Behzad, Christoph Feldmann-Schulz, Bernd Lenz, Lucy Clarkson, Celine Ludwig, Katharina Luttenberger, Simon Völkl, Johannes Kornhuber, Christiane Mühle, Claudia von Zimmermann

**Affiliations:** 1 Department of Internal Medicine 5, Hematology and Oncology, Friedrich-Alexander-Universität Erlangen-Nürnberg (FAU) and Universitätsklinikum Erlangen, 91054 Erlangen, Germany; ali.behzad@uk-erlangen.de (A.B.);; 2 Department of Psychiatry and Psychotherapy, Friedrich-Alexander-Universität Erlangen-Nürnberg (FAU) and Universitätsklinikum Erlangen, 91054 Erlangen, Germanykatharina.luttenberger@uk-erlangen.de (K.L.); christiane.muehle@uk-erlangen.de (C.M.); 3 Department of Addictive Behavior and Addiction Medicine, Central Institute of Mental Health (CIMH), Medical Faculty Mannheim, Heidelberg University, 68159 Mannheim, Germany

**Keywords:** affective disorder, depression, music therapy, non-pharmacological therapy, immune system, translational, randomized waitlist-controlled trial

## Abstract

Background/Objectives: Depression is a prevalent and debilitating illness that significantly affects psychological and physical well-being. Apart from conventional therapies such as psychotherapy and medication, individuals with depression often lack opportunities for activities that are generally perceived as enjoyable, such as music, meditation, and arts, which have demonstrated therapeutic effectiveness. TaKeTiNa music therapy has been employed as a therapeutic intervention for more than two decades. However, there is a notable absence of well-designed clinical trials investigating its antidepressant effects, a gap we aim to address in our current study. Furthermore, shifts in the progression of depression may manifest both psychologically, by influencing emotional states, and physiologically, by leading to alterations in lipid and sphingolipid metabolism, cortisol levels, and immune system function. Our study seeks to analyze the impact of TaKeTiNa music therapy on both levels. Methods: This is a prospective monocentric randomized waitlist-controlled clinical trial. It investigates the influence of TaKeTiNa music therapy on patients with major depression in an outpatient setting. Therefore, interested persons are randomly assigned to two groups, an intervention group or a control group, after completing a screening procedure. The intervention group starts with an eight-week TaKeTiNa music therapy intervention. The waiting group receives the same therapy program after completing the follow-up period. Blood and saliva sampling as well as responses to questionnaires are obtained at specific time points. Discussion: Our study investigates the effects of TaKeTiNa music therapy, a non-pharmacological antidepressant treatment option, on depressive symptoms. We also address functional and causal immunological changes; hormonal changes, such as changes in cortisol levels; and metabolic changes, such as changes in serum lipids and sphingolipids, during the course of depression. We expect that this study will provide evidence to expand the range of treatment options available for depression.

## 1. Background

Depression is one of the most common diseases, with a lifetime risk of up to 20% [1]. It leads not only to psychological but also to physical and social impairments [2,3,4]. Treatment reduces the duration of the disease. Depending on the severity and type of the depressive illness, various forms of treatment are recommended, primarily medication and psychotherapy [1]. However, one-third of patients do not respond to conventional pharmacotherapy, highlighting the need for additional, evidence-based therapies via a multimodal approach. The treatment landscape encompasses non-pharmacological modalities, such as cognitive behavioral therapy [5], mindfulness-based interventions [6], art therapy [7], physical exercise [8], including bouldering (climbing without rope in moderate heights) [9], and the implementation of mental models [9], each offering promising avenues for symptom alleviation and improved quality of life.

In the last two decades, there has been increasing scientific interest in music therapy for mental conditions. Music and music therapy have positive and reproducible effects in different areas, e.g., dementia, insomnia, and anxiety [10,11,12,13,14], and may also improve the tolerability of therapies such as listening to music during intranasal (es)ketamine application [15]. Although there are indications of positive effects on depression [10], these effects appear to be short-term and lack sufficient clinical impact. One reason could be that the studies are usually conducted with very narrow, specific procedures. Interventions such as merely passively listening to classical music or solely rhythmically playing on a drum leave many potential aspects of music therapy untapped, especially because their combination may improve many characteristic symptoms of depression.

TaKeTiNa is a therapeutic modality that harnesses the power of rhythm and movement to foster personal development and overall well-being. Originating from the innovative work of the Austrian musician Reinhard Flatischler, TaKeTiNa music therapy integrates group rhythmic exercises, vocalizations, and bodily movements to induce states of mindfulness, relaxation, and interconnectedness. Participants partake in repetitive rhythmic patterns involving hands, feet, and voice, often in a communal setting, with the goal of facilitating harmonious synchronization of body, mind, and spirit through rhythmic exploration. The name “TaKeTiNa” is emblematic of the rhythmic syllables central to the practice, including “Ta”, “Ke”, “Ti”, and “Na”, which are vocalized and clapped in precise configurations during TaKeTiNa sessions, contributing to its therapeutic impact. Each member of the group is needed for the synchronization of the whole group. Once the group is synchronized, the therapist introduces musical elements in varying degrees of difficulty to let the participants experience their psychological and bodily reactions to outer demands in an immediate way. Through this process, participants become more mindful of their reactions to the outer world. Moreover, through phases of challenging and relaxing elements introduced by the therapist, they learn to deal appropriately with phases of active focus and active relaxation. In this way, TaKeTiNa helps to relearn a healthy response to the changing outer world and an awareness of the inner world while addressing many symptoms of depression, such as social isolation, lack of purpose, adynamia, lack of concentration, and physical inactivity, in therapy sessions.

In the past few years, numerous studies have shown that—among other neurobiological mechanisms of depression [16]—the immune system is involved in the development and course of depression, as well as the therapy resistance of patients [17]. The high number of therapy-refractory patients has led to the need for alternative treatment strategies. The levels of inflammatory cytokines produced by the adaptive immune system, such as tumor necrosis factor (TNF)-alpha, interleukin (IL)-1β, and IL-6, are elevated in patients with depression, whereas the levels of anti-inflammatory cytokines, such as IL-4, IL-10, and transforming growth factor (TGF)-ß, are decreased [18]. Inflammation is assumed to impact neurotransmitter systems and thereby cause depressive symptoms. High inflammation in patients with depression has been shown to be associated with metabolic alterations [19]. Moreover, blocking inflammatory cytokines has been reported to decrease depressive symptoms and reduces the cholesterol concentration in patients with elevated inflammatory markers [19,20]. Many depressed patients exhibit glucocorticoid resistance due to reduced function of the glucocorticoid receptor, which is assumed to be relevant for increased inflammation [21]. Additionally, major depressive disorder, cardiovascular disease, and type 2 diabetes share immune inflammatory alterations [22] and are linked to dyslipidemia. Dyslipidemia, characterized by elevated low-density lipoprotein (LDL) cholesterol, can be associated with chronic cortisol exposure [23]. Prolonged stress and associated dyslipidemia can have a negative impact on health [24]. Heart rate variability (HRV) has been shown to be a sensitive marker for the psychological stress response [25] as well as for individuals with depression during stress [26]. Therefore, the changes described above could be assumed to be linked to altered function of the hypothalamic–pituitary–adrenal axis. In our previous work, we demonstrated that serum lipid levels are associated with depression per se, depression severity, and the prospective course of the disease. We were able to establish the serum lipid levels, particularly the serum LDL concentration, as a biomarker to predict the prospective 20-day short-term course of depression [27]. Treatment with antidepressants had no strong impact on the association between lipid levels and depression severity in our cohort. Thus, independent of antidepressant therapy, immune processes and lipid metabolism might influence each other and lipid metabolism might be an important player in therapeutic resistance in patients with depression [28]. Using the ratio of different immune cells, such as Th1, Th2, and Th17 cells, as surrogate markers, we might be able to better predict the severity of depression and the probable response to therapy. Dysregulation of sphingolipids, a special class of bioactive lipids that are major constituents of membranes, in combination with their metabolizing enzymes related to central ceramide [29,30], has recently gained attention for its role in the pathogenesis and treatment of anxiety and depression in animal models [31,32] and human studies [33,34]. Blood ceramide levels are significantly increased in depressed patients [35,36,37]. We previously showed that the activity of serum acid sphingomyelinase activity, which catalyzes the generation of ceramide, can predict the prospective course of patients [38]. Here, we aim to investigate the interaction between the course of depression after non-pharmacological treatment and serum lipid levels and inflammatory mechanisms.

The goal of this study, which includes a naturalistic cohort, is threefold. First, we hypothesize that participants in the intervention group (IG) receiving 8 weeks of TaKeTiNa music therapy will experience a pre–post decrease in depression. Second, we assess a wide range of potential mediating and moderating factors, such as anxiety, resilience, and mindfulness. Third, we analyze the physiological factor HRV and blood biomarkers, such as serum lipid levels, sphingolipid and corresponding enzyme levels, cytokine levels and immune cell profiles, in relation to changes in depression severity.

## 2. Materials and Methods

### 2.1. Study Design

This is a prospective monocentric randomized waitlist-controlled clinical trial investigating the influence of TaKeTiNa music therapy for patients with major depression in an outpatient group setting on the primary and secondary outcomes of depression.

**Figure 1:**
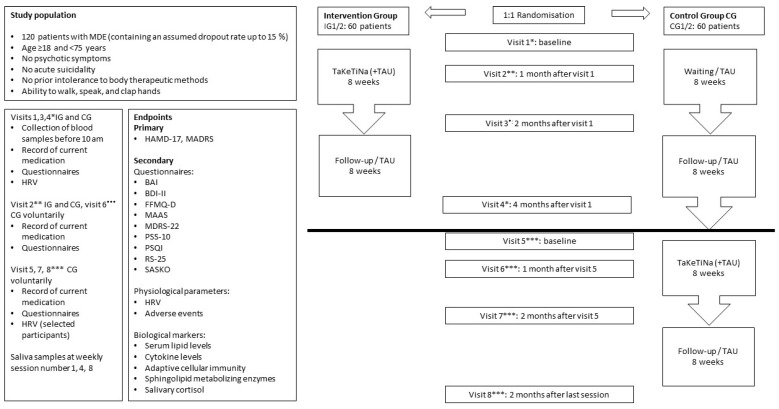
Flowchart for the therapy sequence. BAI: Beck Anxiety Inventory, BDI-II: Beck Depression Inventory, CG: control group, d: day, FFMQ-D: Five Facet Mindfulness Questionnaire, HAMD-17: Hamilton Rating Scale for Depression, HRV: heart rate variability, IG: intervention group with TaKeTiNa music therapy, MAAS: Mindful Attention and Awareness Scale, MADRS: Montgomery–Åsberg Depression Rating Scale, MDE: major depressive episode, MDRS-22: Male Depression Risk Scale, PSS-10: Perceived Stress Scale, PSQI: Pittsburgh Sleep Quality Index, RS-25: Resilience Scale, SASKO: a German questionnaire for social anxiety and deficits in social competence (“Fragebogen zu sozialer Angst und sozialen Kompetenzdefiziten”), TAU: treatment as usual. *, **, and *** indicate the questionnaires and procedures at the visits.

Participants are randomized to either the intervention group (IG) or control group (CG). After the end of the follow-up period, the CG is offered the intervention. To reach the target numbers of participants, there are two consecutive rounds each of intervention (IG1 and IG2) and control (CG1 and CG2) groups. The combined IG1 and IG2 groups are defined as patients who start TaKeTiNa treatment. The combined CG1 and CG2 groups include patients who start with a waitlist (Figure 1).

### 2.2. Objectives

With this study, we aim:

1. To investigate the effects of TaKeTiNa music therapy on depression scores and blood-based parameters, especially LDL cholesterol, in patients with depression.
2. To identify the modifying factors of therapy response, such as anxiety, resilience, and mindfulness.
3. To investigate the relationships between depression-related physiological markers, such as HRV, and biological markers, including immune-system-based biomarkers, lipids, and sphingolipids.

Primary and secondary hypotheses:

Our hypotheses are as follows:

1. TaKeTiNa results in a significant decrease in depression severity due to the intervention (from before visit 1 to visit 3 after 8 weeks of therapy) (combined IG1 and IG2 groups).
2. TaKeTiNa results in significantly stronger pre-to-postintervention depression severity decrease (visit 1 to visit 3) in the IG1/IG2 group compared to the CG1/CG2 group.

Further hypotheses:

- The antidepressive effect of TaKeTiNa postintervention is stable over eight weeks (from visit 3 to visit 4).
- Depressed patients with higher LDL cholesterol also show higher cortisol levels.
- A higher LDL cholesterol level and higher acid sphingomyelinase activity in depressed patients predict greater symptom reduction with (additional) non-pharmacological treatment strategies such as TaKeTiNa.
- TaKeTiNa significantly reduces salivary cortisol levels in depressed patients.
- TaKeTiNa significantly reduces proinflammatory cytokines in depressed patients.
- The HRV at baseline is inversely correlated with the depression severity score and improves during treatment.

### 2.3. Participants

The study is conducted in the city of Erlangen, Germany. Adult outpatient participants with major depression are recruited. The patients are informed via flyers and posters at the hospital as well as via newspaper entries. Study email and phone numbers are provided where interested parties can ask questions and sign up for the study prescreening. A weblink for the prescreening is then sent out to test for eligibility criteria including Patient Health Questionnaire (PHQ-9) as a multipurpose instrument to assess depression severity. Interested persons who prefer a telephone pre-screening to the online link can also choose to do so. If eligible, potential participants are contacted and invited to an in-person meeting with a physician to receive explanations about the study content and procedures and are asked to sign the informed consent form. Participants continue to receive treatment as usual offered by their psychotherapist or medical doctor.

### 2.4. Eligibility Criteria

The inclusion criteria are patient ages between 18 and 75 years and major depressive disorder (Diagnostic and Statistical Manual of Mental Disorders (DSM)-5) [39], which includes recurrent, isolated, and bipolar forms. The exclusion criteria include patients with psychotic symptoms, acute suicidality, prior intolerance to body therapeutic methods, patients who are not able to attend the treatments for organizational reasons, patients who are not able to walk, speak, or clap their hands, and patients with a diagnosis of dementia, including vascular and neurodegenerative forms.

### 2.5. Sample Size

A sample size of 120 was calculated for the primary hypothesis: TaKeTiNa results in a significant pre-to-postintervention decrease in depression severity (combined IG1 and IG2 groups). According to the sample size calculation (one-tailed *t* test, difference between two dependent means, significance level 0.05, power 0.80, G*Power 3.1.9.7, downloaded 2020), 52 participants in the intervention group are sufficient to demonstrate a Cohen’s d of 0.35 and thus even a small effect size. Anticipating a dropout rate of 15% and striving for equal group sizes, our study aims to enroll a total of 120 participants who are evenly distributed between the intervention and control groups.

### 2.6. Randomization/Blinding

Participants are assigned a unique, sequential three-digit number code based on their screening order, with the assignment list securely destroyed after data collection. This proposed study is designed as a wait-list-controlled randomized group pilot study in which patients are randomly allocated to the IG and CG. This randomization process involves sorting participants by sex and age and then alternately assigning them to either the IG or CG to ensure nearly age-matched groups. Raters for rater based interviews are blinded.

### 2.7. Intervention—TaKeTiNa Music Therapy

Participants follow an 8-week protocol with a total of 14 TaKeTiNa units (28 h total) under the supervision of a certified TaKeTiNa teacher. The intervention takes place in a sufficiently large group room at the university hospital. A group may contain a maximum of 35 persons based on previous experience of TaKeTiNa teachers with groups of around 50 individuals.

There is an introductory full day consisting of 3 units. Thereafter, a weekly TaKeTiNa unit is conducted. After 4 weeks, there is another full-day session consisting of 3 units followed by 4 more weekly units. For questions concerning individual participants, at least one medical doctor experienced in psychiatry is always available for the duration of the session. Since the sessions take place at the university hospital, staff in the emergency department of psychiatry are available in case of psychiatric decompensations for the duration of the session or afterwards.

The sessions are generally divided into five stages:

1. Introduction: A brief explanation of the purpose of the session is provided. After that, the teacher introduces the topic (Supplementary Materials List S1) of the session that the participants should pay attention to and to refer to during the rhythmic process.
2. Building the rhythmic foundation: The teacher chooses a TaKeTiNa rhythmic journey among the basic available journeys (Supplementary Materials List S2). Participants gather in a circle. The teacher leads the participants into a repetition of syllables, which facilitates orientation in the music cycle, with a bass drum instrument (Surdo, Remo, CA, USA) in the middle of the circle providing the main rhythm for the process. Then, specific repeating footsteps following the bass drum rhythm are introduced that the participants perform throughout. Last, single or multiple claps are added to introduce a second layer of rhythm that the participants perform. Once the participants are stable in terms of the repeating pattern of talking syllables, stepping, and clapping, the teacher begins the rhythmic journey using a percussive string instrument (Berimbau, Afroton Röttger, Frankfurt am Main, Germany).
3. Rhythmic journey: As the group continues the rhythmic pattern with feet and claps, the teacher moves in the middle of the circle and starts a call response of different melodies and syllables, which the participants sing back as a whole group after they have finished one cycle. The Berimbau provides a subdivision of the main rhythm, making it easier to follow rhythmic melodies in, e.g., the offbeats. The purpose is to use stabilizing and destabilizing rhythms with the voice to increase the flexibility of the participants.
4. Relaxation: As the music slowly fades, the participants are asked to increasingly go inward, with more gentle movements and voices until all movements and sounds subside into an inner feeling of the rhythmic patterns and the participants lay down for approximately 5 min.
5. Integration: Participants and teachers gather in a circle sitting. There is an invitation to share experiences. Here, typical experiences, behavioral patterns, and emotions experienced can be shared and discussed.

### 2.8. Assessment (Primary and Secondary Endpoints)

#### 2.8.1. Data Collection

The data are documented through our study center and can be exported for analysis. Data collection and storage: The data are collected in pseudonymized form on paper and electronically. In all paper and online surveys, participants are encouraged not to provide any data allowing their identification but only their study patient number. The data concerning study findings are digitally stored on the server of the University Hospital Erlangen for 30 years in compliance with data protection regulations. Only the study leaders have access to the reference list (in paper form). The participants can withdraw their consent to participate in the study at any time without giving any reason until the reference list is destroyed after completing the study evaluations. Personal data (e.g., informed consent forms) as well as possible adverse events are stored in paper form in the university hospital in compliance with data protection regulations.

Depression severity and further psychometric scale scores are assessed at regular time intervals (Table 1). The 17-item Hamilton Rating Scale for Depression (HAMD-17) [40] is used to record the primary endpoint, i.e., rater-based depression severity. A trained rater (a physician or medical student assistant) conducts the HAMD-17 interview at the same time as the Montgomery–Åsberg Depression Rating Scale (MADRS) [41]. For further psychometric endpoints, we use the German versions of the Beck Depression Inventory II (BDI-II) [42] for self-evaluation, the Male Depression Rating Scale (MDRS-22) [43,44], and the Beck Anxiety Inventory (BAI) [45]. To additionally assess mindfulness, two scales are utilized: the Mindful Attention and Awareness Scale (MAAS) [46] and the German version of the Five Facet Mindfulness Questionnaire (FFMQ-D) [47,48]. We also determine the effects of the intervention on stress, sleep, and social anxiety by administering the Perceived Stress Scale (PSS-10) [49,50], the Resilience Scale (RS-25) [51], the Pittsburgh Sleep Quality Index (PSQI, [52]), and a German questionnaire for social anxiety and deficits in social competence (“Fragebogen zu sozialer Angst und sozialen Kompetenzdefiziten”, SASKO) [53]. To investigate how personality might influence the response to TaKeTiNa, patients are asked to complete the Big Five Inventory-10 (BFI-10, [54]), a 10-item scale measuring the Big Five personality traits, extraversion, agreeableness, conscientiousness, emotional stability, and openness. All self-evaluation questionnaires are completed online, pseudonymized, at home by the participants. The electronic system enables participants to pause the survey at any point and resume at a later time, allowing them to proceed at their own pace with breaks as needed.

#### 2.8.2. Blood Collection and Analysis and HRV

For biomarkers of the immune system, inflammation, hormones, and sphingolipid parameters, fasted blood samples are collected in serum and EDTA plasma vials at study inclusion, after the last therapy session (or after the two months of waiting), and again two months later (V1, V3, and V4) before 10 a.m. in the morning. Participants are asked to abstain from eating, drinkink, and consuming caffeine or nicotine in the morning prior to the study assessment. After centrifugation, the serum and plasma supernatants are stored as aliquots at −80 °C. A buffy coat from one EDTA vial is used to obtain leukocytes after erythrocyte lysis, which are stored as dry pellets for determination of cellular enzyme activities or stabilized with RNAlater for gene expression analysis via quantitative polymerase chain reaction. The blood samples will be analyzed by measuring the concentration of biomarkers such as growth and immune factors using commercial antibody-based and other detection methods. The activities of enzymes involved in sphingolipid metabolism will be determined via established assays using fluorescently labeled substrates [55,56]. Peripheral blood mononuclear cells are isolated by Ficoll gradient centrifugation and stored at −150 °C for subsequent immunophenotyping. Routine laboratory parameters are assessed at the Central Laboratory of the Universitätsklinikum Erlangen, Germany (DIN EN ISO 15189 accredited; available online under https://www.zentrallabor.uk-erlangen.de/ueber-uns/akkreditierung/ (accessed on 14 April 2024)) and at the Institute of Transfusion Medicine of the University Hospital Erlangen (D-ML-13297-01-00 accredited; available online under https://www.transfusionsmedizin.uk-erlangen.de/fileadmin/einrichtungen/transfusionsmedizin/dateien/2022-11_DAkkS_Urkundenanlage_D-ML13297-01-00.pdf (accessed on 14 April 2024), from separately collected vials. HRV is assessed with the “Elite HRV”^®^ app for mobile systems (ELITE HRV, Asheville, NC, USA) using a Polar H10 ECG® heart rate sensor with chest strap (Polar H10, Kempele, Finland) (Table 1).

### 2.9. Statistical Analysis

The statistical analysis will be conducted using latest version of the SPSS software available at the time of analysis. Group descriptions will be reported showing sociodemographic data as well as relevant primary and secondary outcomes. The nominal significance level will be set at α = 0.05.

### 2.10. Safety

For each participant, there will be three appointments for blood samples (approximately 30–40 mL of blood, i.e., approximately 100 mL in total in four months) and four time points to fill out the online questionnaires. Similar to mindfulness-based practices such as meditation, TaKeTiNa leads to increased awareness. In some cases, such practices can lead to a stronger experience of difficult feelings and thereby exacerbate psychological problems. If study subjects experience an increase in psychological symptoms, they can contact our emergency unit at any time and immediate psychiatric help will be offered. Therefore, we conclude that the potential benefits outweigh the potential risks. People who score more than four on PHQ-9 are asked to call our outpatient care unit for an appointment. If participants show suicidal thoughts in the screening interview (conducted by a medical doctor), treatment options will be discussed with the patients and clinical care will be offered. At least one medical doctor experienced in psychiatry is always available for the duration of the TaKeTiNa units.

Criteria for withdrawal from the study: Each participant has the possibility to withdraw the informed consent. We exclude patients who report suicidal tendencies, severe somatic illness, or admission to the hospital during participation.

## 3. Discussion

We present an 8-week TaKeTiNa music therapy protocol for patients with major depression. In the literature, the positive effects of active musical engagement on mental health outcomes are promising for reducing burden [10,57,58,59,60,61,62].

### 3.1. Choice of Intervention

We chose elements from music therapy because they are an enjoyable practice that does not require dwelling in past hardships or current problems. We more specifically chose TaKeTiNa music therapy for this intervention, as it includes several other techniques that have been proven to mitigate clinical depression, such as physical activity and mindfulness.

Physical activity can effectively reduce symptom burden in patients with depression [63]. Therefore, TaKeTiNa music therapy may have an additional effect on depressive symptoms.

Mindfulness-based techniques have been shown to reduce symptom burden in patients with depression [64] and are an essential part of the TaKeTiNa music therapy process. Participants, while “just doing music”, are constantly made aware of their body’s movement, tension, emotions, reactions to outer stimuli, etc., as every aspect can be a distraction as the challenges in music and the focus increase. Organically, over time, people learn to pay attention to their thoughts, feelings, tension in the body, and other influencing factors so that they can choose to let go. To measure the potential increase in awareness, we will use the MAAS and the FFMQ.

Not being able to address the demands of the environment adequately is both a sign of depression and a driver of the disease in daily life [65]. As a result, people with depression are in a state of chronic stress or tension, which is reflected by decreased HRV [26,66]. TaKeTiNa music therapy leads the group and each individual through a ride of relaxation to reach the limit of their capabilities, to overwhelm them before they return to a state of relaxation. The participants experience thoughts and feelings associated with these states, such as success and pride, sadness and disappointment, resistance, denial, thoughts of superiority or inferiority, and so on. Through these cycles and the states they provoke, participants can relearn how to be in full activity, focus, and arousal and then let go into a deep, mindful relaxation and, through this, reteach the body to reduce its sympathetic overactivity. This could help individuals cope with perceived stress. To assess these aspects, we use both a questionnaire for resilience (RS-25) and a questionnaire for perceived stress (PS-10), while also measuring the HRV over time.

### 3.2. Frequency and Timespan of Therapy

For the frequency and timespan of the intervention, we chose an intensive introduction on a Saturday followed by eight weekly intervention meetings on Wednesdays with a second more intensive Saturday meeting four weeks after the start of the intervention. In the published literature, weekly sessions seem not to be inferior to daily interventions [57] and are much more feasible both in a clinical setting and one with limited resources but also in this particular group of patients, as adynamia and overstimulation may lead to drop outs.

### 3.3. Expected Benefits

To our knowledge, this study represents the first investigation of TaKeTiNa music therapy using a high-quality study design. With the Center for Immune Therapy in Germany (DZI), we have a unique opportunity to understand the functional and causal immunological changes that are thought to influence and cause depression [17,18,20]. For this reason, we plan to measure several metabolic markers that are associated with depression, such as serum lipids [27], hormones such as cortisol, and the level of cellular function, as well as the ratios of different immune cells and their production levels of cytokines and their ability to be stimulated in vitro. We will also assess the novel aspect of masculine depression (scale MDRS) characterized by specific traits and burdens with possibly different underlying mechanisms [43,67,68].

The trial will help to clarify the effects of TaKeTiNa therapy on depression symptoms and markers and, beyond that, provide insight into its use in medicine in general [69]. By monitoring biomarkers and immune cells over time, we aim to identify biological predictors of both disease severity and resistance to conventional therapy. We will contribute to the fundamental understanding of the mechanisms of depression and immunity.

## Figures and Tables

**Table 1:** Assessment times. * indicates before and after the first, fourth, and last evening session, ** indicates that participation is voluntary for the waiting group during the intervention phase following their waiting time.

Visits	Prescreening	Screening	V1,3,4	V2, V6 **	V5,7,8 **
PHQ-9 or telephone interview	x				
Inclusion/exclusion criteria		x	x		
Informed consent		x			
Recording of					
- medical history and life events			x		x
- current medication			x	x	x
- HAMD-17			x		
- MADRS			x		
- BAI			x	x	x
- BDI-II			x	x	x
- MAAS/FFMQ-D			x	x	x
- MDRS-22			x	x	x
- PSS-10			x	x	x
- RS-25			x	x	x
- SASKO			x		x
- PSQI			x		
- blood sample			x		
- saliva sample *					
- heart rate variability			x		x

The table shows assessment times and data collected. V: visit. See legend of Figure 1 for abbreviations.

## Data Availability

The data are available upon request.

## Supplementary Materials

The following supporting information can be downloaded at: https://www.mdpi.com/article/10.3390/jcm13092494/s1, List S1: Topics for the TaKeTiNa units, List S2: Journeys for the TaKeTiNa units.

## List of Abbreviations

BAI	Beck Anxiety Inventory
BDI-II	Beck Depression Inventory II
BFI-10	Big Five Inventory-10
CG	control group
DSM	Diagnostic and Statistical Manual of Mental Disorders
DZI	Center for Immune Therapy in Germany
FFMQ-D	Five Facet Mindfulness Questionnaire
HAMD-17	Hamilton Rating Scale for Depression—17
HRV	heart rate variability
IG	intervention group
IL	interleukin
LDL	low-density lipoprotein
MAAS	Mindful Attention and Awareness Scale
MADRS	Montgomery–Åsberg Depression Rating Scale
MDE	major depressive episode
MDRS-22	Male Depression Risk Scale
PHQ-9	Patient Health Questionnaire
PSQI	Pittsburgh Sleep Quality Index
PSS-10	Perceived Stress Scale—10
RS-25	Resilience Scale—25
SASKO	Fragebogen zu sozialer Angst und sozialen Kompetenzdefiziten
TAU	treatment as usual
TGF	transforming growth factor
TNF	tumor necrosis factor
