# Supplementary material for: TaKeTiNa Music Therapy for Outpatient Treatment of Depression: Study Protocol for a Randomized Clinical Trial
Source: J Clin Med. 2024 Apr 24;13(9):2494. doi: 10.3390/jcm13092494 (PMC11084329; doi:10.3390/jcm13092494)
Supplement: Supplementary file 1 [file jcm-13-02494-s001.zip › jcm-2963403-supplementary.pdf]

**TaKeTiNa-Music Therapy for outpatient treatment of depression: study protocol for a randomized clinical trial**

## Supplemental Material

List 1: Topics for the TaKeTiNa units

The topics discussed during the TaKeTiNa process are not presented in any particular order or hierarchy but are commonly encountered themes. The TaKeTiNa facilitator, based on their assessment of the group dynamics or individual processes, selects a topic at the beginning of each session (as outlined in Methods: Intervention stage 1). This approach allows participants to focus on the chosen topic throughout the session, enhancing their awareness of its relevance and how they can integrate it into their daily lives. During the integration phase at the conclusion of the session (Methods: Intervention stage 5), the chosen topic is revisited, and participants, as well as facilitators, briefly share their experiences and thoughts.

Some of the key topics explored during the TaKeTiNa process include:

- Embracing instances of falling out of rhythm as a natural element of the learning journey
- Encouraging participants to detach from the rhythm at any point
- Supporting individual learning paces
- Techniques for achieving rhythmic synchronization with the floor
- Exploring primal rhythms and embodying them
- Addressing inner evaluative thoughts that may surface during the process
- Engaging in simultaneous perception exercises
- Exploring the rhythmic voice and its connection to internal and external communication
- Investigating the interplay between auditory perception, verbal expression, and bodily movement
- Cultivating trust in the supportive ground
- Sensing the nuances of steps, such as dropping, stomping, or lightly touching the floor, while maintaining rhythmic weight shifts
- Understanding the balance between muscle tension and relaxation
- Exploring energetic connections between the soles and crown of the feet
- Utilizing inner visualization techniques in rhythm exploration
- Observing the link between stress and boredom in rhythmic experiences
- Reshaping the linear perception of time
- Reconnecting with primal rhythmic sensations while lying down
- Recognizing obstructive behavioral patterns and strategies to avoid being ensnared by them
- Balancing chaos and order to achieve relative coordination
- Harnessing self-organizing capabilities
- Cultivating the willingness to be supported by the group
- Embodying individual and collective experiences in harmony
- Practicing maintaining eye contact while staying in rhythm with others
- Engaging in multifaceted vocal rhythm experiences, including Mantra, soul talk, and collaborative vocalizations
- Encouraging learning beyond the constructs of praise and blame
- Addressing feelings of self-doubt and mistrust in oneself and others
- Managing fears related to loss of control, surrender, and conformity to specific rhythmic structures
- Coping with apprehensions about potential criticism for mistakes
- Exploring and overcoming disabling behavioral patterns.

List 2: Journeys for the TaKeTiNa units:

The rhythmic components, known as "Journeys" in TaKeTiNa, provide the foundational rhythmical structure for the specified process. The suffixes in each Journey name mirror the recurrent syllables uttered by participants throughout the session (refer to Methods: Intervention stage 2). The initial segment of each Journey signifies the introduction of demanding or stimulating rhythmic elements to be superimposed onto the foot patterns via clapping.

For instance, in a sequence like "Offbeat Ti with MuSanGaLa," the designation indicates that a clap occurs off the beat at precise midpoints between every four steps (Mu, San, Ga, La). Meanwhile, variations such as "3 over 2" signify that the feet move in a cycle of two beats (e.g., two four-measure beat cycles), while the hands follow a cycle of three beats (e.g., three two-measure beat cycles), facilitating the creation of complex polyrhythms for enhanced rhythmic and musical challenges.

The selection of each Journey by the TaKeTiNa instructor is based on the group's collective rhythmic and musical proficiency, as well as the facilitator's assessment of the group's collective needs to delve deeper into the process (e.g., by introducing more demanding or stabilizing elements). Beside the gradient of difficulty, ranging from easy to challenging, each Journey, owing to its unique blend of rhythmic elements, elicits various effects, such as propelling the group forward (instigating an unconscious increase in speed or beats per minute if unregulated), grounding, tugging, heightening awareness, among others.

The list of Journeys entails:

- Offbeat Ti with MuSanGaLa
- Offbeat Ma with GaLaGa
- Offbeat La with MuSanGaLa
- Tresillo over MuSanGaLa
- 2 over 3 with GaMaLa
- 3 over 2 with MuSanGaLa, LaGa, GaLaGa
- 2 over 5 with GaMaLaTaKi
- 5 over 2 with GaLaTa
- 3 over 4 with LaGa
- 4 over 3 with TaKeTi
- 3 over 9
- Beat over Tresillo
